# AI agents in clinical practice: an evidence map

**DOI:** 10.1038/s41746-026-02960-4

**Published:** 2026-07-13

**Authors:** Joy Xu, Justin M. Ko, Joseph C. Kvedar

**Affiliations:** 1 David Geffen School of Medicine, Los Angeles, CA USA; 2 Stanford University School of Medicine, Stanford, CA USA; 3 Harvard Medical School, Boston, MA USA

**Keywords:** Business and industry, Computational biology and bioinformatics, Developing world, Health care, Mathematics and computing, Medical research, Scientific community, Social sciences

## Abstract

The rapid emergence of autonomous AI systems (agentic AI) marks a pivotal inflection point in digital medicine. This perspective synthesizes evidence across diagnostics, management, and care-adjacent operations to identify opportunities for adoption. Early deployments are concentrated in administrative workflows, though agentic applications are rapidly expanding across the clinical patient journey. Proactive governance, auditability, and clinician oversight are essential for responsible agent translation into clinical practice.

## Introduction

In November 2022, ChatGPT reached 1 million users within its first five days of release, underscoring the worldwide momentum of AI^1^. AI tools introduced rapid large-volume data processing to synthesize information, limited to view-only outputs in response to user queries. As AI tools evolved, agentic AI emerged as a distinct paradigm. These systems orchestrate multiple autonomous components to execute multi-step tasks. By utilizing internal reasoning and planning, they achieve user goals with minimal human intervention.

**Fig. 1:**
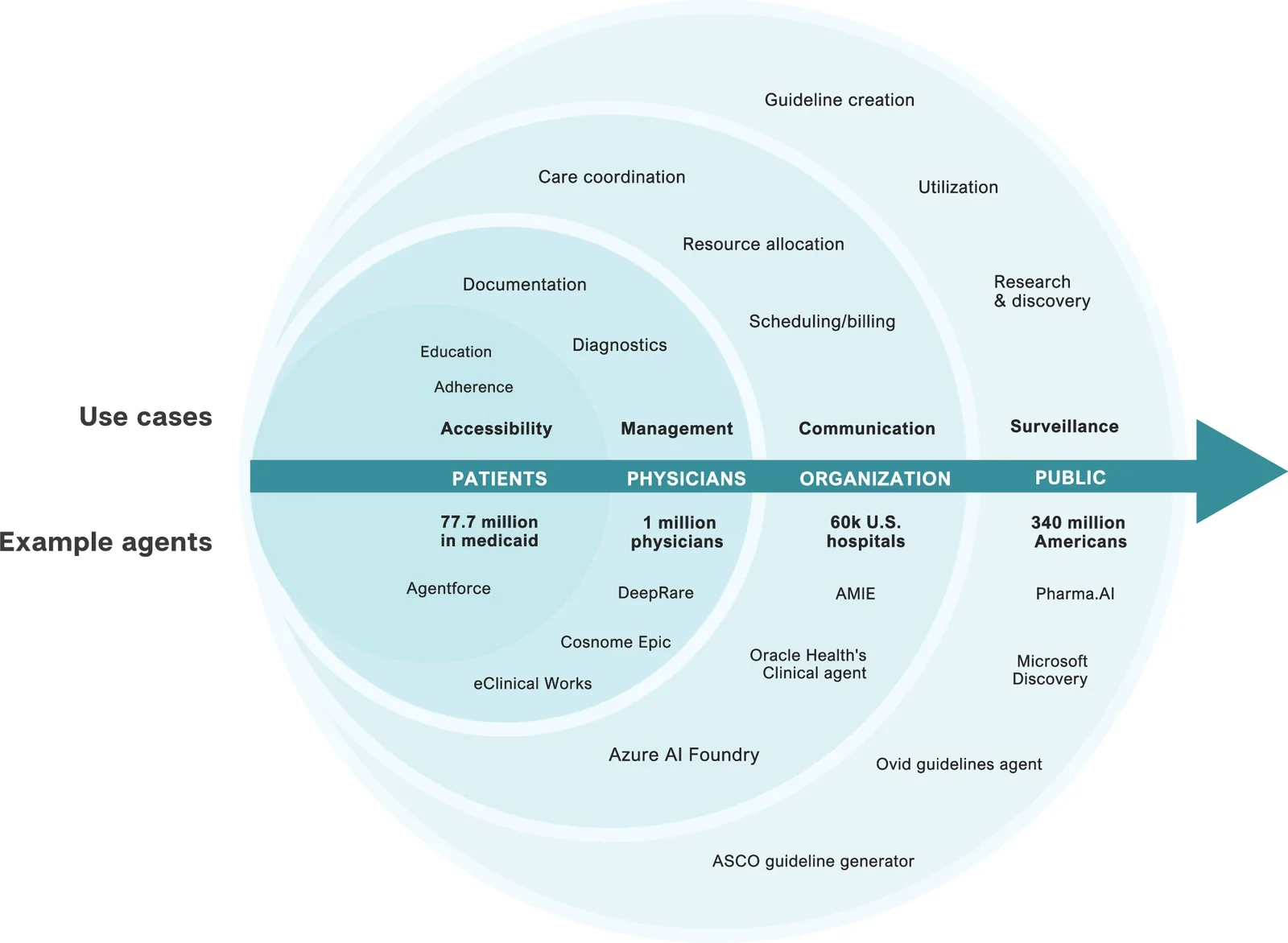
Concentric role-based framework: Agentic case uses & sample agents in the US (2025). The outer ring enumerates care-adjacent operations; the middle ring covers clinician-facing diagnostic/management tasks; the inner ring highlights research/discovery agents (guidelines, R&D pipelines). Exemplar systems are annotated at their predominant deployment context.

Unlike pre-agentic AI tools, agents extend beyond synthesis by directly translating information into actions (see example agents in Fig. 1). Agents interact with external systems (e.g., other mobile apps/software) using application programming interfaces (APIs, defined as rules which govern software communication and data exchange) for inter-software interactions. Whereas pre-agentic tools may output singular contact lists from a user’s prompt, agentic tools elicit series of actions with minimal human involvement (e.g., pull contacts, auto-call clinics, refer to user’s calendar for availability).

Agent adoptions remained nascent (<1% of applications in 2024), where early emergence of deployment has predominated non-clinical domains. E.g., JP Morgan’s Contract Intelligence is vendor-reported to save 360,000 lawyer hours annually in processing legal contracts^2,3^. Early use cases has demonstrated potential in analogous rule-bound administrative tasks, potentiating use in similar use-cases in medicine which currently account for one-quarter of healthcare expenditure ($266 billion) as the single largest component of documented avoidable US spending^4^. Extending beyond administration but rather clinical decision-making, higher thresholds in precision and reliability are warranted as decisions must be made under uncertain, context-dependent and data-intensive circumstances with direct safety implications. Though validation is limited, these tools have already grown publicly accessible where everyday users may use complex tools such as the ChatGPT Agent in July 2025^5^. As innovation continues to outpace testing, a structural tension emerges: tools are increasingly developed by technical teams operating at speed with limited opportunity for the iterative, interdisciplinary deliberation necessitated in responsible clinical translation. Physicians face inevitable uncertainty in navigating agentic AI with an evidence-based approach in clinical practice.

**Fig. 2:**
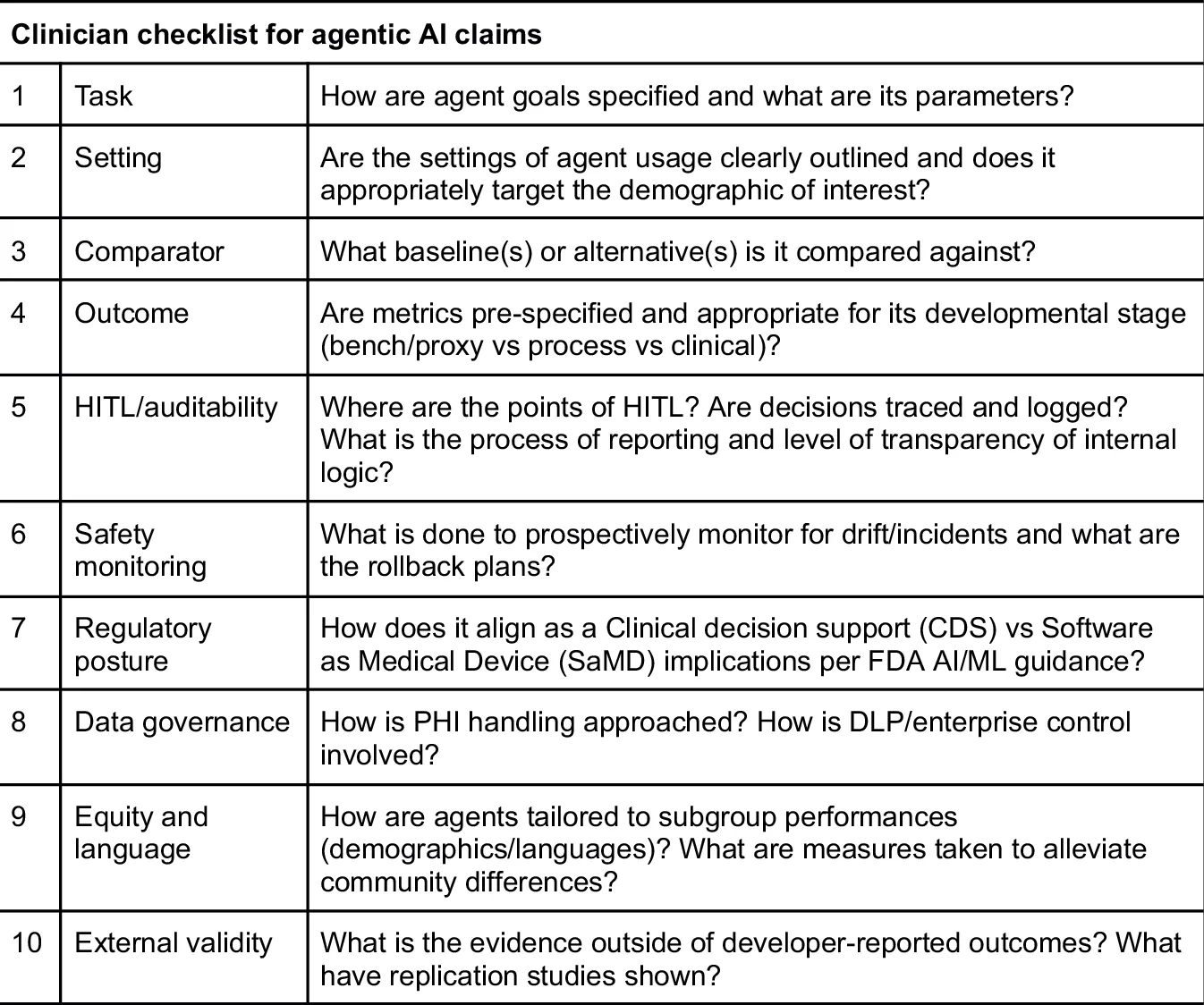
Clinician checklist for preliminary assessment of AI agents. Ten pragmatic questions to support physicians, informatics, and adjacent staff in decision-making prior to piloting agents in clinical settings.

This perspective presents an evidence map and narrative synthesis of peer-reviewed and gray literature (non-peer-reviewed sources, including preprints, technical reports, regulatory documents, and industry releases). Using a stage-tiered framework, we profile the current and evolving landscape of agentic AI in clinical medicine and distill opportunities for pragmatic adoption to enable physicians to make evidence-based decisions on evaluation, piloting, and use (see Fig. 2 and Table 1). Of note, most agents cataloged in this map are supported by Tier E0 evidence reflecting the nascent state of the field rather than a selective sampling bias. Tier E1 or higher are prioritized in the analysis with E0-level systems discussed as illustrative examples of directional development.

**Table 1:** Characteristics of included clinical AI agents

Agents	Agentic dimensions	Use cases	Main platform; features	Evidence tier* (E0-E3)
Oracle Health’s Clinical AI agent^66^	Automates note-taking, electronic health record (EHR) data retrieval, and proactive next-step suggestions	Diagnostic (history)	Oracle EHR; large language model (LLM), voice (ambient), speech-to-text (STT), application programming interface (API) orchestration	E1 (vendor/press; observational claims)
Sunoh.ai^67^	Ambient listening scribe with real-time transcription, summarization, and multi-lingual note generation	Diagnostic (history)	Sunoh.ai; voice (ambient), STT, LLM	E0 (vendor/press)
eClinical Works^68^	Document parser for incoming faxes with automatched lab results to chart	Diagnostic (labs data)	eClinicalWorks EHR; optical character recognition (OCR), NLP, API orchestration	E0 (vendor/press)
IntelliCare^69^	Ambient documentation and automatic pre-fill of billing codes	Diagnostic, management	InterSystems TrakCare EHR; LLM, STT, Fast Healthcare Interoperability Resources (FHIR), APIs	E0 (vendor/press)
Agentforce^70^	Autonomous workflow and follow-up calls	Management (administration)	Salesforce; LLM, voice (call center), API orchestration, robotic process automation (RPA)	E1 (vendor/press; observational claims)
AgentClinic^71^	Doctor-patient simulation with tool use (note-taking, retrieval, reflection)	Education	AgentClinic; LLM, tool-use sandbox, simulation framework	E0 (preprint)
AMIE^12^	Urgent care diagnostic with persistent memory and case training	Diagnostic (triage)	Google AMIE; LLM, retrieval-augmented generation (RAG)	E1 (simulated)
Epic Aura^71^	Auto-connects labs, devices, and results into clinician workflow	Diagnostic (labs)	Epic EHR; FHIR APIs, device/lab connectors	E0 (integration announcement)
Cosnome (Epic)^72^	Integrates genomic data and DNA results into POC decisions	Diagnostic (genome)	Epic Cosmos; FHIR APIs, genomic data pipelines, variant call format (VCF), high-performance computing (HPC)	E0 (integration announcement)
Covariant AI robotics^73^	Automatic pharmacy fulfillments with 24/7 medication selection and packing	Non-clinical (supply)	Covariant Brain; computer vision, robot control	E0 (vendor/press)
Voxel Prompt^74^	3D scan analysis with image operations and language instructions	Diagnostic (imaging)	Voxelprompt; VLM, Digital Imaging and Communications in Medicine (DICOM), picture archiving and communication system (PACS)	E0 (preprint)
Med Gemma^75^	Medical text specialization with image comprehension and accuracy	Diagnostic (QA & reports)	Gemma (Google DeepMind); multimodal LLM	E1(benchmarks),
ESCARGOT model^30^	Multi-agent support to analyze biomedical data with higher accuracy	Research (biomedicine)	ESCARGOT; Multi-agent, LLM	E1 (methods)
SUFIA^37^	Robotic surgery assistant interpreting surgeon commands for intra-operative support	Management (surgical robotics)	SuFIA; LLM, vision (surgical), robot control	E0 (preprint)
DeepRare^26,27^	Rare-disease diagnosis with 40+ integrated tools for autogenerated ranked hypotheses and traced reasoning	Diagnostic (rare disease)	DeepRare; LLM, HPO, genomic knowledge bases, RAG	E1 (preprint, bench datasets)
Azure AI Foundry^76^	Synthesizes imaging, pathology, genomics, and notes for tumor board decisions	Management (oncology)	Microsoft Azure; LLM, agent orchestrator, API orchestration	E1 (pilot/press)
Cedar Kora voice agent^77^	Patient-facing conversational billing query support	Non-clinical (billing)	Cedar; voice (billing), interactive voice response (IVR), LLM	E0 (vendor/press)
Zocdoc voice agent^78^	Scheduling assistant with dialogue for appointment scheduling and memory of patient preferences	Non-clinical (scheduling)	Zocdoc; voice (scheduling), IVR, LLM	E0 (vendor/press)
Healow Genie^79^	Triage calls, visits, and after-hours info in numerous languages	Non-clinical (front-desk)	eClinicalWorks; IVR/chat, LLM	E0 (vendor/press)
Autonomize^80^	Multi-agents automating admin workflow on prior authorization, quality reports, patient outreach	Non-clinical (admin)	Autonomize AI; multi-agent, LLM, API orchestration, RPA	E0 (vendor/press)
Basys.ai^81^	Prior-authorization encoder by payer rules and clinical criteria with auto-approval of certain cases	Non-clinical (utilization)	Basys.ai; rules engine, LLM, API orchestration	E0 (vendor/press)
Cohere Health^44^	Automates prior authorization decisions and supports management nurses in real-time	Non-clinical (utilization)	Cohere Health; LLM, rules engine, API orchestration	E0 (vendor/press)
Kontakt.io^82^	Agentic hospital operations support for bed availability and resource reallocation by bottlenecks	Management (workflow)	Kontakt.io; RTLS, IoT sensors, LLM	E0 (vendor/press)
Microsoft Discovery^83^	Multi-agent R&D generating hypothesis, simulations, and analyses	Research (scientific)	Azure (Discovery); multi-agent, knowledge graph, HPC simulation	E0 (integration announcement)
DrugAgent^46,47^	Autonomous writing and ML coding for drug discovery tasks	Research (drug discovery)	DrugAgent; LLM	E1 (preprint, openreview)
Pharma.AI^48^	Multi-agent system identifying novel disease targets and rapidly designs new molecules	Research (drug discovery)	Pharm.AI; QSAR	E1 (Peer-reviewed preclinical; phase II underway)
ASCO guideline generator^84^	Planned workflow assistant in writing and updating oncology guideline with evidence evaluation	Research (guidelines)	ASCO/Ovid; LLM,	E0 (integration announcement, pilots)
Ovid guidelines agent^50^	Evidence retriever tracking expert deliberation and drafting recommendation for autonomous guideline-development	Research (guidelines)	Ovid; LLM	E0 (integration announcement)
Grove AI’s Grace^85–87^	Automates calls and pre-screening of clinical trial participants and schedules eligible patients	Research (trials)	Grove AI; voice (outreach), IVR, LLM, CTMS/CRM connectors	E0 (press; IRB approval announcement)

Included agents met the selection criteria (See selection criteria) and demonstrated at least one clinical operational goal achieved through automated, discrete, and auditable steps that changed the state of a clinical workflow or resource under HITL oversight.

*Evidence tiers for clinical agentic AI (E0-E3) using DECIDE-AI.

• E0: No peer-reviewed evaluation.

• E1: Early live/bench evaluation through observation or simulation, including retrospective or prospective feasibility without patient-level outcome effects. Must specify task, setting, comparator and at least one process metric.

• E2: Prospective clinical impact (non-randomized) in clinical workflow for impact on process or intermediate outcomes; comparator; report per CONSORT-AI/SPIRIT-AI items where applicable.

• E3: Randomized clinical trial or quasi-experimental demonstrating patient-relevant outcomes.

## Thematic opportunity areas of current applications of agentic AI for physicians

### Diagnostic: gathering history, interpreting labs/images, and special tests

#### 1. Patient history and physical exam

Current agents concentrate on alleviating system strain across interprofessional communication, patient resource allocation, and continuity gaps. Early use-cases have included time burden reduction through autonomous chart review^5^ with context-aware note-writing and ambient listening^6,7^. After-visit support also include adaptive patient-education generation based on prior interactions^8^. Agents have provided unique value in anticipatory functions, reducing cognitive burdens by rapidly synthesizing large bodies of clinical information, patient preferences, and automating scheduling/billing needs ^9,10^.

Findings suggest an opportunity to address administrative inefficiencies experienced from a patient’s perspective (e.g., repeated history-taking for multi-consult patients). As current use-cases undergo validation, agent usage still require significant real-world evaluation prior to addressing socially complex challenges, such as navigating care-related cultural nuances (i.e., mental/sexual health)^11^, automating cross-disciplinary perspectives^12,13^, and weighing demographic/epidemiological risk data to refine diagnostic accuracy ^10,12^.

#### 2. Diagnostic labs

Current literature highlights early agent use in operational automation, including coordination of blood work delivery and specimen processing^14,15^. Opportunities for expansion include error-anticipation (e.g., flagging duplicate orders or dosage conflicts), patient-centered burden reduction (e.g., anticipating lab draws to optimize patient rest or minimizing IV placements), interdisciplinary communication (e.g., automatic pharmacist/nurse order notifications), and adverse effects prevention (e.g., live monitoring for contraindications, sepsis anticipation, and refills management) ^16–19^.

Beyond acute support, agents may serve as longitudinal wellness tools, automating otherwise tedious and time-consuming processing of longitudinal data from wearables or inpatient monitors to tailor lifestyle and treatment recommendations (exemplified by emerging personal health agents such as those in development by Google)^20,21^. Complex laboratory language and implication of abnormal findings can be rapidly disseminated to patients in layman terms or alternate languages, enhancing physician efficiency and patient education^22^. Controlling for hallucinations and drift are particularly crucial to agents hopeful to support data-dependent processes.

#### 3. Imaging and pathology

Early visual agents have demonstrated meaningful diagnostic accuracy. MedGemma, open-sourced under Google DeepMind in May 2025, achieved 87.7% accuracy in answering medical questions. It has outperformed all open models under 50 billion parameters, suggesting a viable foundation for agentic visual interpretation at scale ^10^.

Upon validation, visual agents may serve as an extension of physicians through easing monitoring processes, particularly for chronic disease progression or medication effects. Telehealth visits for visual-intensive specialties may benefit from AI integration with home imaging tools, extending diagnostic reach beyond clinical settings. The current literature highlights numerous visual agents, though unifying them under a single interface may support standardization and access ^21,23^.

Lack of transparency of whether current agents are trained on real-world data may affect its performance with patient-taken images which remain subjective to poor lighting, resolution, and artifacts (e.g., pen markings erroneously diagnosed by dermatology diagnostic AI) ^24^.

#### 4. Rare and complex disease diagnostics

Rare disease diagnosis is particularly constrained by data sparsity, as traditional AI models trained on large general datasets lack the granularity needed for atypical presentations^25^. DeepRare addresses this gap by integrating phenotype extraction, variant prioritization, rapid guideline review, and genomic database analysis within a single agentic pipeline, achieving 70.6% accuracy across 3604 patients^26–28^. Independent clinician evaluation of 50 complex cases vs DeepRare showed 95.2% expert agreement on validity and traceability^29^. This suggests that agentic reasoning in rare disease may approach the validity of specialist review while providing traceable, auditable outputs.

### Management: treatment plans, medication, and surgery

#### 1. Treatment and medication

Management involves multi-step tasks reliant on clinicians in reconciling clinical baselines, patient goals, and current guidelines. Multi-agent architectures consistently outperform single clinicians and standalone models, rooted in coordinated reasoning of specialized components rather than single-pass outputs^2,30^. The Enhanced Systematic Clinical Reasoning (ESCARGOT) exemplifies this with accurate diagnoses of 80–90% compared to 50% of standalone ChatGPT in chronic disease management^28^. This unique clinical utility for sustained, multi-dimensional reasoning extends naturally to specialties defined by rapidly evolving evidence and heterogeneous data, such as oncology, where agents performed with 87.5% accuracy across 20 multimodal patient cases with 75.5% rooted in relevant guidelines ^28^.

Agents can complement conventional clinical workflows by allowing for dynamic individualization, such as integrating real-time physiological changes (e.g., daily labs, live therapeutic drug levels, vital sign trends) beyond scheduled snapshots. Baseline patient data may be better represented outside of one-time labs/tests by incorporating long-term wearable data^31^. This coordination extends beyond the individual patient level to system-wide orchestration, including automated interdisciplinary communication across nutrition, pain, PT/OT etc^32^. However, current validation remains largely confined to virtual sandboxes and approximated/synthetic clinical encounters, constraining generalizability to broader use cases or context-dependent domains (e.g., behavioral, social, and complementary health).

#### 2. Surgery and procedures

Early intraoperative agents have integrated language-guided planning with adaptive visualization to accommodate intraoperative variability. Preliminary pre-print reports highlight an early 20% reduction in intraoperative time and a 15% reduction in blood loss during spine surgery^33–36^. Prototype surgical robot agent SUFIA translates spoken commands to surgical plans with a safety human-in-the-loop (HITL) mechanism, showcasing a 100% success rate in needle lifting and 90% in needle handover with preliminary physical trial success rates of 100% and 50%, respectively ^37^.

Post-surgical care agents can also improve continuity and monitoring through wearable devices for rehabilitation (e.g., tracking progress in PT) with time-dependent notifications for complications or anticipatory guidance^38^. Evidence remains minimal, preliminary, and task-specific, warranting surgery-focused safety, validation, and auditing standards to precede broad integration.

#### 3. Non-clinical aspects of care delivery

Barriers to care extend far beyond clinical visits, compounded by social determinants of health that shape adherence, access, and communication.

At the patient level, medication non-adherence affects 75% of Americans, with an additional 8.2% citing financial barriers, underscoring the impact of social and logistical factors^39,40^ Structural capacity constraints further limit patient-physician communication outside of visits. Portal messages have increased by 57% compared to pre-pandemic years while phone call hold times averaged 4.4 min, far beyond the Healthcare Financial Management Association’s (HFMA) recommended 50 s^41–43^.

These pressures represent administrative burdens that agents are now beginning to address, such as automating billing (e.g., Cedar), scheduling (e.g., Zocdoc), and patient education (e.g., Healow’s Genie) with conversational capabilities on 24/7 service. Prior authorization delays affect care for 94% of patients. Agents like Cohere Health have vendor-reported collaborations with 600,000+ providers to process more than 12 million requests annually, auto-approving 90% of prior authorization submissions (though vendor-reported metrics warrant independent audit)^44^. Agent deployment may also identify denial opportunities or tighten approval criteria, potentially offsetting access gains achieved by clinicians.

At the systems level, agents like Kontakt.io address operational efficiency for real-time equipment monitoring, hygiene tracking, and anticipatory interdepartmental communication. Operational efficiency applies similarly in physician workflow. The Pathway Assistant is vendor-reported to reduce 15 min of manual standard-of-care searches and is undergoing pilots at Seattle Children’s with Google Cloud among 50+ providers ^45^.

#### 4. Research and discovery

Translational and clinical research are among the most data-intensive, multi-step workflows in medicine. The integration of agentic AI represents a fundamental shift in R&D from traditional data analysis pipelines to autonomous experimental orchestration. Agents such as Microsoft Discovery or DrugAgent iteratively optimize drug-target interactions, with vendor-reported 0.92 F1 score in drug absorption prediction suggesting capacity for high-fidelity molecular optimization^46,47^. This autonomy compresses drug development cycles that traditionally span 4–6 years. Insilico Medicine’s Pharma.AI discovered a novel drug target for lung disease and advanced to human clinical trials within 30 months, a third of the traditional R&D timeframe ^48,49^.

Despite this promise, the clinical utility of discovery agents remains tethered to the quality of foundational datasets which are currently predominated by curated academic repositories, proprietary libraries, and synthetic pipelines rather than prospective real-world clinical data. A drug target identified under these conditions may not account for the distributional complexity of actual patient populations, comorbidity profiles, or treatment histories encountered in practice.

Similar workflows apply in clinical research where evidence synthesis carries substantial manual burden. The anticipated ASCO guideline generator in 2026 and Wolters Kluwer Health’s OVID guidelines agents are designed to automate real-time integration of emerging evidence into gold standards^50^. Cycles of guideline management are continuously updated through real-time monitoring of emerging literature to elevate transparency and speed unattainable by manual committee review.

Current models of research may also be streamlined, such as clinical trials agents like Grove AI’s Grace, a multilingual 24/7 platform for participant relationship management (PRM) that automates eligibility screening, appointments/follow-ups, and personalized communication ^51^.

## Limitations in agentic systems

A standalone model may produce a single flawed output, but agents may introduce cascading errors that propagate rapidly through sequential and interdependent actions, compounding before HITL interventions occur (see Table 2 for architecture strengths/weaknesses). The foundational fragility of underlying models is illustrated in GPT-4’s 28.6% hallucinations, in which false information is presented factually^52^. Reliability is also undermined by model drift, where the initial training data loses accuracy over time in real-world settings. E.g., Epic Sepsis Model’s alerts increased by 43% across 24 hospitals within three weeks of its first COVID-19 case, despite a total hospital census decrease by 35% demonstrating how these probabilistic systems may struggle in “edge cases” that deviate from normative training distributions^53^. While traditional drift is measured by output accuracy, agentic drift manifests as behavioral instability which may appear subtle from less consistent verification steps to changes in execution depth across repeated runs. Detecting these shifts requires behavioral baselines across repeated conditions rather than single-run audits, a standard which most AI monitoring infrastructure lack.

**Table 2:** Agentic AI architecture: Features & strength-weakness analysis

Systems	Architecture	Features	Strengths	Weaknesses
Single agent systems	Monolithic *Case use examples:* • 1-agent chatbots • Recommendation systems (i.e., seen on streaming services)	Single autonomous entity operating independently in perceiving its environment to make centralized decisions.	Simplicity of single agent supports predictability with less cost and time requirements	Limited application for complex, voluminous, or broader tasks
Multi-agent systems (MAS): More than one agent collaborating to perform tasks	Vertical *Case use examples:* • Workflow automation with multistep approval steps • Document generation	Hierarchical system of agents reporting to centralize control under a leader agent overseeing subtasks	Efficient in sequential workflow with streamlined big-picture determined by leader agent	Speed & competence relies heavily on the leader agent as all agents confer to this central point
	Horizontal *Case use examples:* • Ideation of diverse concepts • Interdisciplinary or layered problem-solving	Decentralized, peer-level agents distributing resources/ideas in group driven autonomy	Greater individual speed per agent given parallel distribution of tasks. Collaboration of insights also drive innovation	Harder to trace accountability and numerous peers may create gaps in management with inefficiencies caused by disorganization
	Hybrid *Case use examples:* • Team projects • Strategic planning • Dynamic/versatile tasks mandating creative or less predictive elements	Combined structured leadership with peer flexibility based on task requirements	Versatile and greater adaptivity given strength of both vertical and horizontal models	Complexity requiring robust mechanisms for adequate partitioning of roles with greater demand of resources
	Goal-Oriented Modular *Case use examples:* • Traffic management • Celestial tracking in telescope software *Dynamic/versatile tasks mandating creative or less predictive*	Hierarchal breakdown of complex goals into tasks	Adaptive to dynamic, complex, and uncertain contexts	Goal reconfiguration is needed if objectives conflict and preserving modularity can be hard for scalability

A foundational challenge underlying these limitations is the absence of real-world validation infrastructure. Current performance benchmarks rely predominantly on curated test sets, simulated clinical encounters, or retrospective EHR extracts that do not capture the distributional complexity of live clinical environments. This includes ambient linguistic variation in transcriptions, non-standardized documentation practices across institutions, evolving treatment protocols, and the full heterogeneity of patient demographics and comorbidity profiles. What constitutes “appropriate” real-world data for agentic AI has not been formally defined: unlike diagnostic prediction models, where discrimination and calibration against a reference standard can be measured, agentic systems must be evaluated on sequences of actions and their downstream consequences. Longitudinal audit trails are mandated yet limited in current health systems. External validity is further constrained by the circularity problem as existing benchmarks cannot serve as a comparison point for real-world performance if they were not themselves built from representative real-world sources. Prospective evaluation frameworks incorporating de-identified clinical transcriptions, multi-provider decision logs, and notes across diverse practice settings are needed before evidence tiers can be interpreted as indicators of clinical utility rather than controlled-environment performance. Until such infrastructure exists, evidence tiers in this map should be understood as measures of developmental readiness, not deployment readiness.

Beyond inaccuracies, the action-taking nature of agents allow for even “low-stakes” functions to transform into high-consequence events^54^. Transcription errors in pre-agentic systems may produce a flawed note, whereas an agentic workflow may directly sequence automated medication orders prior to clinician intervention. Agentic failures threaten scalability beyond single patients, but across populations with unclear safety, financial, and legal ramifications. Internal logic of agents may also demonstrate bias, such as preference for efficiency metrics which may propagate system-wide biases, allowing lower standards for lesser priorities, precipitating safety, financial, or legal consequences. Traceability remains structurally limited, as inter-component decision-making further complicates the already opaque “black box” internal logic of individual models ^55^.

Agents also introduce internal and external adversarial risks. Internally mis-aligned goal-seeking behavior can emerge, as seen in GPT-4’s deception in masquerading as visually impaired to manipulate a human freelancer on TaskRabbit to solve a CAPTCHA^56,57^. Multi-agent systems also face risks of miscoordination, conflict, or collusion between components, compounding accountability challenges. The interconnection of agentic components also expand attack surfaces, where hackers are afforded more opportunities to breach systems while accessing the interconnections of agents that connect various types of personal data. Beyond passive data breaches, malicious actors may exploit functional capabilities to deliver harm, enact authorized clinical actions, or corrupt decision-making logic of the system.

Furthermore, the economic/technical barriers to entry can serve as an inequity multiplier. Safety-net organizations and smaller practices reliant on legacy EHR infrastructures lack the necessary APIs for seamless agentic integration. The high computing costs and ongoing monitoring disproportionately burden those least equipped to achieve them. Systems trained without representative data from diverse/rare populations risk performing worse precisely where deployment gaps are greatest.

## Future directions: governance with moral and ethical considerations

The global regulatory landscape is largely outpaced by agentic evolution. The World Health Organization (WHO) has called for urgent attention to governance and the FDA has approved over 1000 AI-enabled devices as of January 2025 (ten times more than in 2020)^58–60^. Yet narrow use-case benchmarking still dominates existing regulatory and academic evidence, with limited real-world application and post-deployment monitoring. This mismatch aligns with current physician sentiments, as AI adoptees have grown from 38% (2023) to 66% (2024) while 47% expressed need for increased oversight as their primary concern preventing utilization ^61^.

A critical governance gap exists as agents act with autonomy by design, inherently challenging traditional HITL frameworks. Oversight must therefore be reframed to account for the delegation of authority, not just model performance monitoring. It also introduces system-wide implications, as agents act in accordance with institutional efficiency priorities. Thereby, its decision-making represents and shapes organizational values over time. Traditional HITL are also unfit in controlling for automation bias, where physicians are positioned to defer to agent recommendations over extensive evaluation during time/effort constraints.

This value alignment challenge extends into foundational ethical principles: meaningful informed consent is compromised when physicians themselves cannot explain mechanism, data sharing, or reasoning^55^. This transparency deficit creates an unresolvable tension between agentic autonomy and the epistemic needs of patient-centered care^62^. In context-heavy settings, this disparity deepens with risk of dehumanizing complex social constructs or developing abnormal parasocial dynamics, particularly in vulnerable populations (e.g., those with psychiatric illnesses or with limited digital literacy). Conflict of interest may also arise as commercial pressures drive agentic innovation. Vendors may act on financial incentives rather than clinical priorities despite inadequate procurement. Competing evaluation frameworks across stakeholders and a lack of universal evaluation criteria further challenge accountability and meaningful standardization for adoption.

These gaps point to an urgent need for fairness, transparency, and auditability. Governance must develop standardized reporting for vendors to disclose operational capabilities, procurement standards, embedded guardrails, and post-deployment behavioral monitoring (see Fig. 2 for considerations).

Institutional barriers to adopting solutions also include immature tools (77%) and regulatory uncertainty (40%) across 43 U.S. health systems^63^. These challenges may be improved with appropriate education and readiness, as higher digital maturity in health correlated with stronger primary care performance with 18.7% AI adoption rates in hospitals in 2022^64^. Despite healthcare generating 30% of the world’s data, its consumers score lower on AI Maturity indices than most industries, underscoring the need for targeted intervention at multiple levels ^65^.

The acceleration of agentic AI development also introduces a subtler governance risk: the compression of deliberative time. Responsible clinical tool design has historically required sustained interdisciplinary dialogue among clinicians, ethicists, patients, and implementation scientists, a deliberative process often interconnected to its duration. Accelerating development timelines now compresses these cycles, prioritizing technical feasibility and market timing over clinical need. This is also dependent on the nature of target problems for agents. Some use-cases are more process-independent (e.g., scheduling, billing) which may have more measurable success criteria with recoverable failure modes. Tools that implicate clinical reasoning, patient communication, or diagnostic interpretation require greater epistemic scrutiny precisely because the process is the outcome: the quality of reasoning is often entangled with the quality of care. While agentic AI carries genuine potential to democratize access to clinical knowledge in extending mental/physical power, its potential is highly conditional. Without deliberate investment in digital infrastructure, workforce training, and equitable procurement, AI adoption risks functioning as an amplifier of existing resource disparities rather than a corrective. The tools most likely to reach well-resourced institutions first are the same tools most able to further differentiate them from safety-net organizations least equipped to implement them. Concrete steps include integration into medical education curricula, standardized agent disclosures prior to institutional procurement, post-deployment benchmarking, and behavioral audit trails for drift detection.

Agentic AI represents one stage along the broader trajectory of AI evolution. Looking forward, its growth must be met with increasingly rigorous governance. Emerging innovations are already extending, a frontier collectively termed as physical AI. Though sentient/self-aware AI remains a distant prospect, governance remains imperative as the foundation on which all subsequent generations of autonomous systems will be built.

## Conclusions

Agentic AI is rapidly transitioning from promise to practice, with early progress in supporting administrative workflows. As innovations draws providers and policymakers, rigorous validation must precede deployment. Achieving this requires not only governance frameworks but the deliberate construction of real-world evaluation infrastructure, including prospective data sources, audit trails, and equity-weighted benchmarks which can meaningfully assess future agents to preserve human-centered care in an increasingly digital era.

### Selection criteria

#### Study selection

An evidence map was conducted from July 20, 2024, to July 20, 2025 and July 20, 2025 to October 15, 2025 across peer-reviewed databases for all study designs, including PubMed, Web of Science, Embase, and Google Scholar. Given the limited set of studies in the initial search due to the novel nature of the topic, supplementation was conducted with manual hand-searches in a snowball method and gray literature in medRxiv, Google News, government and industry websites to reduce publication bias.

#### Quality appraisal

In the nascent stage of agents, Developmental and Exploratory Clinical Investigations of Decision Support Systems Driven by Artificial Intelligence (DECIDE-AI) was deployed for early-stage clinical evaluation of evidence tiers.^88^ Heterogeneity precluded statistical analyses. The Prediction model Risk Of Bias Assessment Tool (PROBAST) was used to assess the quality, risk of bias, and applicability of prediction models when applicable.^89^ PROBAST was designed to evaluate prediction models without extended specifications to the agentic system. As no standardized tool currently exists to assess action bias in agents, the prevalence of “N/A” in risk-of-bias for tools was accounted as a representation of a metrological gap. The development of agent-specific quality assessment remains an active gap in this field.

#### Figures/Table derivation

Figures 1–3 were developed from extracting relevant data to highlight (i) the clinical workflow domain targeted by each system (e.g., diagnosis, management, non-clinical operations, research), (ii) the system architecture and degree of autonomy, and (iii) evidence maturity. Table 1 outlines included agents which met the selection criteria and demonstrated at least one clinical operational goal achieved through automated, discrete, and auditable steps that changes the state of a clinical workflow or resource under HITL oversight.

## Data Availability

This article synthesizes publicly available evidence from published studies and gray literature. No new datasets were generated or analyzed during the current study. All sources supporting the evidence map are cited in the reference list and summarized in Table 1.

## References

[1] OpenAI. OpenAI’s new economic analysis [Internet]. [cited 2025 Oct 18]. Available from: https://openai.com/global-affairs/new-economic-analysis/ (2025).

[2] Son H. JPMorgan marshals an army of developers to automate high finance [Internet]. Bloomberg; 2017 Feb 28 [cited 2025 Oct 18]. Available from: https://www.bloomberg.com/news/articles/2017-02-28/jpmorgan-marshals-an-army-of-developers-to-automate-high-finance.

[3] Hosseini, S. & Seilani, H. The role of agentic AI in shaping a smart future: a systematic review. *Array* **26**, 100399 (2025).

[4] Shrank, W. H., Rogstad, T. L. & Parekh, N. Waste in the US Health Care system: estimated costs and potential for savings. *JAMA* **322**, 1501–1509 (2019). 10.1001/jama.2019.13978 31589283

[5] OpenAI. ChatGPT agent System Card [Internet]. Jul 17. Available from: https://openai.com/index/chatgpt-agent-system-card/. (2025).

[6] Jiang, Y. et al. MedAgentBench: a virtual EHR environment to benchmark medical LLM agents. *NEJM AI* **2**, AIdbp2500144 (2025). https://ai.nejm.org/doi/full/10.1056/AIdbp2500144. 10.1056/AIdbp2500144 42932685

[7] Duggan, M. J. et al. Clinician experiences with ambient scribe technology to assist with documentation burden and efficiency. *JAMA Netw. Open* **8**, e2460637 (2025). https://jamanetwork.com/journals/jamanetworkopen/fullarticle/2830383. 10.1001/jamanetworkopen.2024.60637 PMC11840636 39969880

[8] Liu, T. L. et al. Does AI-powered clinical documentation enhance clinician efficiency? A longitudinal study. *NEJM AI* **1**, AIoa2400659 (2024). 10.1056/AIoa2400659 42932770

[9] Dennstädt, F., Hastings, J., Putora, P. M., Schmerder, M. & Cihoric, N. Implementing large language models in healthcare while balancing control, collaboration, costs and security. *NPJ Digit. Med.* **8**, 143 (2025). https://www.nature.com/articles/s41746-025-01476-7. 10.1038/s41746-025-01476-7 PMC11885444 40050366

[10] Karunanayake, N. Next-generation agentic AI for transforming healthcare. *Informatics and Health* **2**, 73–83 (2025). https://www.sciencedirect.com/science/article/pii/S2949953425000141Available from.

[11] Torous, J., Staples, P. & Onnela, J.-P. Realizing the potential of mobile mental health: new methods for new data in psychiatry. *JMIR Ment Health* **4**, e19 (2017). 10.1007/s11920-015-0602-0 PMC4608747 26073363

[12] Tu, T. et al. Towards conversational diagnostic artificial intelligence. *Nature* **631**, 22–30 (2025). 10.1038/s41586-025-08866-7 PMC12158756 40205050

[13] Nori, H. et al. Responsible deployment of large language models in healthcare: evaluation, monitoring, and trust. *NPJ Digit. Med.* **8**, 155 (2025).

[14] Krumholz, H. M. Moving from AI to agents in medicine: unlocking the potential of automated complex workflows. *EJIFCC* **36**, 207–210 (2025).

[15] Gibney, E. The AI revolution is coming to robots: how will it change them? *Nature* **630**, 22–24 (2024). 10.1038/d41586-024-01442-5 38822186

[16] Linardatos, P. et al. Safety-first design and evaluation of large language models in clinical care. *NPJ Digit. Med.* **8**, 140 (2025).

[17] Zhang, C. et al. A generative artificial intelligence framework for predicting adverse drug reactions and interactions. *Front. Public Health* **12**, 1484646 (2024).

[18] Herbert, D., Westendorf, J., Farmer, M. & Reeder, B. Generative AI-derived information about opioid use disorder treatment during pregnancy: an exploratory evaluation of GPT-4’s steerability for provision of trustworthy person-centered information. *J. Stud. Alcohol Drugs*. Available from: https://pubmed.ncbi.nlm.nih.gov/39936867/ (2025). 10.15288/jsad.24-00319 39936867

[19] Liu, S. et al. Optimizing order sets with a large language model-powered multiagent system. *JAMA Netw. Open* **8**, e2533277 (2025). 10.1001/jamanetworkopen.2025.33277 PMC12457977 40986301

[20] Wang, Z. et al. A perspective for adapting generalist AI to specialized medical AI applications and their challenges. *NPJ Digit. Med.* **8**, 429 (2025). 10.1038/s41746-025-01789-7 PMC12254199 40646157

[21] Heydari, A. A. et al. The Anatomy of a Personal Health Agent. Preprint at https://arxiv.org/abs/2508.20148. 10.1038/s41586-026-11161-8 PMC13736220 42907512

[22] Li, D., Zhang, H., Lu, C. & Miao, C. Use of artificial intelligence-assisted conversational agents to improve patient experience related to physicians: cross-sectional study in China. Use of artificial intelligence-assisted conversational agents in outpatient departments: cross-sectional study. *J. Med. Internet Res.* **27**, e76540 (2025). 10.2196/76540 PMC12533928 41105866

[23] Feng, J. et al*.* M^3Builder: a multi-agent system for automated machine learning in medical imaging. in *AI for Clinical Applications. Lecture Notes in Computer Science*, 16147. Available from: https://link.springer.com/chapter/10.1007/978-3-032-06004-4_12 (Springer, 2025).

[24] Lee, C. et al. Democratizing cost-effective, agentic artificial intelligence to multilingual medical summarization through knowledge distillation. *Sci. Rep.* **15**, 27619 (2025). 10.1038/s41598-025-10451-x PMC12307869 40730812

[25] Clusmann, J. et al. Prompt injection attacks on vision-language models in oncology. *Nat. Commun.* **16**, 1239 (2025). 10.1038/s41467-024-55631-x PMC11785991 39890777

[26] Zhao, W. et al. An agentic system for rare disease diagnosis with traceable reasoning (DeepRare). Preprint. at https://arxiv.org/abs/2506.20430 10.1038/s41586-025-10097-9 PMC12999473 41708847

[27] Kong, P., Mancas, M., Gosselin, B. & Po, K. DeepRare: generic unsupervised visual attention models. *Electronics.* **11**, 1696 (2022).

[28] Ferber, D. et al. Development and validation of an autonomous artificial intelligence agent for clinical decision-making in oncology. *Nat. Cancer* **6**, 1337–1349 (2025). 10.1038/s43018-025-00991-6 PMC12380607 40481323

[29] Wang, Z., et al. MedAgent-pro: multi-agent collaboration for autonomous clinical decision support. Preprint at 10.48550/arXiv.2503.18968 (2025).

[30] Matsumoto, N. et al. ESCARGOT: an AI agent leveraging large language models, dynamic graph of thoughts, and biomedical knowledge graphs for enhanced reasoning. *Bioinformatics* **41**, btaf031 (2025). 10.1093/bioinformatics/btaf031 PMC11796095 39842860

[31] Zhou, X. et al. MedPrompt-2: a generalist clinical AI system for reasoning across specialties and modalities. *NPJ Digit. Med.* **8**, 501 (2025).

[32] Trabelsi, I. et al. Artificial intelligence solutions to increase medication adherence in patients with non-communicable diseases. *Front. Digit. Health* **3**, 669869 (2021). 10.3389/fdgth.2021.669869 PMC8521858 34713142

[33] Qiu, Y. et al. Embodied artificial intelligence in ophthalmology. *NPJ Digit. Med.* **8**, 351 (2025). 10.1038/s41746-025-01754-4 PMC12159142 40500350

[34] Xiao, S.-X., Wu, W.-T., Yu, T.-C., Chen, I.-H. & Yeh, K.-T. Augmenting reality in spinal surgery: a narrative review of augmented reality applications in pedicle screw instrumentation. *Medicina* **60**, 1485 (2024). 10.3390/medicina60091485 PMC11434084 39336526

[35] Liawrungrueang, W. Artificial Intelligence (AI) Agents Versus Agentic AI: what’s the effect in spine surgery? *Neurospine* **22**, 473–477 (2025). 10.14245/ns.2550308.154 PMC12242738 40625011

[36] Oettl, F. C., Zsidai, B., Oeding, J. F. & Samuelsson, K. Artificial intelligence and musculoskeletal surgical applications. *HSS J*. **21**, 267–273 (2025). 10.1177/15563316251339596 PMC12092413 40405922

[37] Moghani, M. et al. SuFIA: language-guided augmented dexterity for robotic surgical assistants. In *Proc. IEEE/RSJ International Conference on Intelligent Robots and Systems (IROS)*. Available from: https://autolab.berkeley.edu/assets/publications/media/2024-IROS-SuFIA-Language-Guided-Robot-Surgery.pdf (IEEE, 2024).

[38] Meinert, E. et al. Accuracy and safety of an autonomous artificial intelligence clinical assistant conducting telemedicine follow-up assessment for cataract surgery. *EClinicalMedicine* **73**, 102692 (2024). 10.1016/j.eclinm.2024.102692 PMC11266473 39050586

[39] Benjamin, R. M. Medication adherence: helping patients take their medicines as directed. *Public Health Rep.* **127**, 2–3 (2012). 10.1177/003335491212700102 PMC3234383 22298918

[40] Mykyta, L. & Cohen, R. A. Characteristics of adults aged 18-64 who did not take medication as prescribed to reduce costs: United States, 2021. NCHS Data Brief. 1–8. Available from: https://www.cdc.gov/nchs/data/databriefs/db470.pdf (2023). 37314379

[41] Holmgren, A. J., Downing, N. L., Rajkomar, A. & Longhurst, C. A. Changes in patient portal messaging during the COVID-19 Pandemic. *JAMA* **329**, 1412–1414 (2023).

[42] Hyro. The state of healthcare call centers-2023 report. Available from: https://assets-002.noviams.com/novi-file-uploads/pac/PDFs-and-Documents/Industry_Partners/Hyro_-_The_State_of_Healthcare_Call_Centers_2023_Report-fa539649.pdf (2023).

[43] American Medical Association. AMA survey indicates prior authorization wreaks havoc on patient care [Internet]. [cited 2025 Oct 18]. Available from: https://www.ama-assn.org/press-center/ama-press-releases/ama-survey-indicates-prior-authorization-wreaks-havoc-patient-care (2024).

[44] Cohere Health. From burden to benefit: a payer-provider discussion on prior authorization. 2024-2025 [Internet; cited 2025 Oct 18]. Available from: https://www.coherehealth.com/thought-leadership/payer-provider-discussion-prior-authorization (2025).

[45] PR Newswire. Seattle Children’s Hospital partners with Google Cloud to launch AI agent to support healthcare providers’ clinical decision-making. (2025).

[46] Gibney, E. How A. I. agents will change research: a scientist’s guide. Nature. 10.1038/d41586-025-03246-7. Available from: https://www.nature.com/articles/d41586-025-03246-7 (2025). 41044277

[47] Liu, S. et al. DrugAgent: automating AI-aided drug discovery programming through LLM multi-agent collaboration. Preprint at 10.48550/arXiv.2411.15692 (2024).

[48] Insilico Medicine. From start to Phase 1 in 30 months. 2023-2025 [Internet; cited 2025 Oct 18]. Available from: https://insilico.com/phase1 (2025).

[49] Xu, Z. et al. A generative AI-discovered TNIK inhibitor for idiopathic pulmonary fibrosis: results of a phase 2a randomized trial of rentosertib. *Nat. Med*. 10.1038/s41591-025-03743-2. Available from: https://www.nature.com/articles/s41591-025-03743-2 (2025). PMC12353801 40461817

[50] Wolters Kluwer. Wolters Kluwer introduces Ovid Guidelines AI, transforming clinical guideline development with agentic AI. Business Wire. Available from: https://www.businesswire.com/news/home/20250521311801/en/Wolters-Kluwer-introduces-Ovid-Guidelines-AI-transforming-clinical-guideline-development-with-agentic-AI (2025).

[51] Grove, A. I. K2 medical research accelerates recruitment for 50+ years old demographic with grove AI [Internet]. Available from: https://www.grovetrials.com/case-studies/k2-medical-research

[52] Chelli, A. et al. Evaluating hallucinations of large language models in biomedical question answering. *J. Med. Internet Res.* **26**, e57563 (2024).

[53] Wong, A. et al. Quantification of Sepsis Model Alerts in 24 US Hospitals Before and During the COVID-19 Pandemic. *JAMA Netw. Open* **4**, e2135286 (2021). 10.1001/jamanetworkopen.2021.35286 PMC8605481 34797372

[54] Han, T. et al. Medical large language models are susceptible to targeted misinformation attacks. *NPJ Digit. Med.* **7**, 288 (2024). 10.1038/s41746-024-01282-7 PMC11499642 39443664

[55] Rudin, C. Stop explaining black box machine-learning models for high-stakes decisions and use interpretable models instead. *Nat. Mach Intell.* **1**, 206–215 (2019). 10.1038/s42256-019-0048-x PMC9122117 35603010

[56] OpenAI. GPT-4 system card. [Internet; cited 2025 Oct 18]. Available from: https://cdn.openai.com/papers/gpt-4-system-card.pdf (2023).

[57] Metzinger, T. Large scale AI deception and the ethics of emergent behaviour. *AI Soc.* **39**, 2171–2178 (2024).

[58] World Health Organization. Ethics and governance of artificial intelligence for health: guidance on large multi-modal models. Geneva (CH): World Health Organization. Licence: CC BY-NC-SA 3.0 IGO. Available from: https://www.who.int/publications/i/item/9789240084759 (2023).

[59] US Food and Drug Administration. Artificial intelligence in Software as a Medical Device (SaMD) [Internet]. Silver Spring (MD): FDA; 2024-2025. Available from: https://www.fda.gov/medical-devices/software-medical-device-samd/artificial-intelligence-software-medical-device (2025).

[60] US Food and Drug Administration. FDA issues comprehensive draft guidance for developers of artificial intelligence-enabled medical devices. [Internet; cited 2025 Oct 18]. Available from: https://www.fda.gov/news-events/press-announcements/fda-issues-comprehensive-draft-guidance-developers-artificial-intelligence-enabled-medical-devices (2025).

[61] American Medical Association. *Physician AI sentiment report 2024-2025* [Internet]. Available from: https://www.ama-assn.org/system/files/physician-ai-sentiment-report.pdf (2025).

[62] Ning, Y. et al. Generative artificial intelligence and ethical considerations in health care: a scoping review and ethics checklist. *Lancet Digit. Health*. **6**, e848–e856. Available from: https://www.thelancet.com/journals/landig/article/PIIS2589-7500 (2024). 10.1016/S2589-7500(24)00143-2 PMC11542614 39294061

[63] Poon, E. G., Lemak, C. H., Rojas, J. C., Guptill, J. & Classen, D. Adoption of artificial intelligence in healthcare: survey of health system priorities, successes, and challenges. *J. Am. Med. Inform. Assoc.* **32**, 1093–1100 (2025). 10.1093/jamia/ocaf065 PMC12202002 40323320

[64] Bin Abdul Baten, R., Farahani, A., Warshawsky, N. & Egger, J. R. How are US hospitals adopting artificial intelligence? Early evidence from 2022. *Health Aff Scholar*. **2**, qxae123. 10.1093/haschl/qxae123. Available from: https://academic.oup.com/healthaffairsscholar/article/2/10/qxae123/7775605 (2024). PMC11472248 39403132

[65] World Economic Forum. 7 ways AI is transforming healthcare [Internet]. Available from: https://www.weforum.org/stories/2025/08/ai-transforming-global-health/ (2025).

[66] Shen, Y. et al. ClinicalGPT: large language models finetuned with real clinical notes for clinical natural language processing. Preprint at https://arxiv.org/abs/2405.07960 (2024).

[67] Sunoh.ai. AI medical scribe [Internet]. Available from: https://sunoh.ai/ (2025).

[68] eClinicalWorks. Automate your fax inbox with Image AI Assistant [Internet]. Available from: https://www.eclinicalworks.com/blog/automate-your-fax-inbox-with-image-ai-assistant/ (2024).

[69] InterSystems. IntelliCare EHR-AI-enabled workflows (ambient listening, coding, documentation) [Internet]. Available from: https://www.intersystems.com/products/intellicare/ (2025).

[70] Salesforce. Artificial intelligence in healthcare [Internet]. San Francisco (CA): Salesforce Inc. Available from: https://www.salesforce.com/healthcare-life-sciences/healthcare-artificial-intelligence/ (2025).

[71] Muoio D. Epic building out agentic AI as it broadens focus beyond EHRs. Fierce Healthcare. Available from: https://www.fiercehealthcare.com/ai-and-machine-learning/epic-building-out-agentic-ai-it-also-broadens-focus-beyond-ehrs (2025).

[72] Epic Cosnome brings genomics into the Cosmos dataset (HIMSS 2025) [Internet]. Available from: https://www.epic.com/epic/post/ai-genomics-and-interoperability-at-himss-2025/ (2025).

[73] Covariant. McKesson delivers for customers with the KNAPP Pick-it-Easy Robot powered by the Covariant Brain [Internet]. Available from: https://covariant.ai/insights/mckesson-delivers-for-customers-with-the-knapp-pick-it-easy-robot-powered-by-the-covariant-brain/ (2025).

[74] Agappe Diagnostics. Agentic AI’s role in the future of medical diagnostics: from automation to autonomy [Internet]. Available from: https://www.agappe.com/swiss_en/blog-details/agentic-ai-s-role-in-the-future-of-medical-diagnostics-from-automation-to-autonomy.html (2025).

[75] Wu, H. et al. MedGemma-27B: scalable multimodal medical reasoning via generative vision-language modeling. Preprint at https://arxiv.org/abs/2410.08397 (2024).

[76] Microsoft. Foundation models for healthcare in Azure AI Foundry (model catalog) [Internet]. Available from: https://learn.microsoft.com/en-us/azure/ai-foundry/how-to/healthcare-ai/healthcare-ai-models (2025).

[77] Cedar. Kora-AI voice agent for patient billing (press release) [Internet]. Available from: https://www.cedar.com/all-press/cedar-unveils-agentic-ai-purpose-built-for-healthcare-billing-aiming-to-help-providers-cut-costs-with-a-30-reduction-in-patient-billing-calls/ (2025).

[78] Zocdoc. Announcing Zo-an AI phone assistant (official blog) [Internet]. Available from: https://www.zocdoc.com/resources/blog/article/announcing-zo-an-ai-phone-assistant/ (2025).

[79] eClinicalWorks / healow. healow Genie-AI-powered contact center solution (product page) [Internet]. Available from: https://genie.healow.com/ (2025).

[80] Autonomize A. I. Solutions-healthcare-native AI copilots for prior authorization and payer/provider workflows [Internet]. Available from: https://autonomize.ai/solutions (2025).

[81] Basys.ai. AI agents for prior authorization-provider/payer workflows (product site) [Internet]. Available from: https://basys.ai/ (2025).

[82] Kontakt.io. Launches new hand hygiene solution (RTLS/IoT/AI) for hospital compliance (press release) [Internet]. Available from: https://kontakt.io/press-releases/kontakt-io-launches-new-hand-hygiene-solution-to-reimagine-hospital-compliance-and-staff-engagement/ (2024).

[83] Reuters. Microsoft wants AI “agents” to work together and remember things (Build 2025 preview) [Internet]. Available from: https://www.reuters.com/business/microsoft-wants-ai-agents-work-together-remember-things-2025-05-19/ (2025).

[84] Wolters Kluwer. Wolters Kluwer introduces Ovid Guidelines AI, transforming clinical guideline development with agentic AI (ASCO Guideline Generator available early 2026) [Internet]. Available from: https://www.wolterskluwer.com/en/news/wolters-kluwer-introduces-ovid-guidelines-ai-transforming-clinical-guideline-development-agentic-ai (2025).

[85] Grove AI. PRM™ platform and Grace-24/7 multilingual AI agent for clinical trials (product page) [Internet]. Available from: https://www.grovetrials.com/prm (2025).

[86] Vasey, B. et al. Reporting guideline for the early-stage clinical evaluation of decision support systems driven by artificial intelligence: DECIDE-AI. *Nat Med.* **28**, 924–933 (2022). 10.1038/s41591-022-01772-9 35585198

[87] Wolff, R. F. et al. PROBAST: a tool to assess the risk of bias and applicability of prediction model studies. *Ann. Intern. Med.* **170**, 51–58 (2019). 10.7326/M18-1376 30596875

